# The Efficacy of miR-20a as a Diagnostic and Prognostic Biomarker for Colorectal Cancer: A Systematic Review and Meta-Analysis

**DOI:** 10.3390/cancers11081111

**Published:** 2019-08-03

**Authors:** Laura Moody, Svyatoslav Dvoretskiy, Ruopeng An, Suparna Mantha, Yuan-Xiang Pan

**Affiliations:** 1 Division of Nutritional Sciences, University of Illinois at Urbana-Champaign, Urbana, IL 61801, USA; 2 Department of Kinesiology and Community Health, University of Illinois at Urbana-Champaign, Urbana, IL 61801, USA; 3 Department of Kinesiology and Community Health, Division of Nutritional Sciences, University of Illinois at Urbana-Champaign, Urbana, IL 61801, USA; 4 Carle Physician Group, Carle Cancer Center, Carle Foundation Hospital, Urbana, IL 61802, USA; 5 Department of Food Science and Human Nutrition, Division of Nutritional Sciences, and Illinois Informatics Institute, University of Illinois at Urbana-Champaign, Urbana, IL 61801, USA

**Keywords:** microRNA, miR-20a, colorectal cancer, diagnosis, prognosis

## Abstract

*Background*: MicroRNAs have altered expression levels in various diseases and may play an important role in the diagnosis and prognosis of colorectal cancer (CRC). *Methods*: We systemically reviewed and quantitatively synthesized the scientific evidence pertaining to microRNA-20a (miR-20a) as a CRC biomarker. A keyword and reference search in PubMed yielded 32 studies, in which miR-20a was measured in feces, serum, or tumor tissue. Data were extracted from a total of 5014 cancer cases and 2863 controls. *Results*: Twenty out of 21 relevant studies found that miR-20a was upregulated in CRC patients compared to controls. Meta-analysis revealed a pooled miR-20a fold change of 2.45 (95% CI: 2.24–2.66) in CRC patients versus controls. To estimate sensitivity and specificity of miR-20a as a diagnostic biomarker of CRC, a pooled area under the receiver operating characteristic curve (AUROC) was calculated (0.70, 95% CI: 0.63–0.78). The prognostic capacity of miR-20a was assessed using hazard ratios (HRs) for the overall survival (OS). The meta-analysis estimated the pooled HR for OS to be 2.02 (95% CI: 0.90–3.14) in CRC patients with high miR-20a expression. *Conclusions*: miR-20a may be a valid biomarker for CRC detection but may not be a strong predictor of poor prognosis in CRC.

## 1. Introduction

Colorectal cancer (CRC) is the third most common cancer worldwide and is responsible for approximately 50,000 deaths per year in the United States alone. If detected in the early stages, adenomas can be surgically removed and prognosis is favorable, with the five-year survival rate reaching 90%. However, once the cancer metastasizes to the lymph nodes and distant organs, the five-year survival rate drops to 60% and 10%, respectively [1]. At more advanced stages, chemoradiation therapy (CRT) may become a viable neoadjuvant or adjuvant treatment option. For instance, in stage IV resectable colon cancer, neoadjuvant chemotherapy may be beneficial in identifying candidates for surgery and improving three-year disease-free survival [2]. Furthermore, there is currently no effective method to predict patient response. For rectal cancer, a “watch and wait” approach is commonly employed after surgery or CRT to assess cancer recurrence and determine whether more aggressive treatment should be administered [3,4]. Novel biomarkers are needed to expedite tumor detection and improve patient stratification for intervention purposes.

Currently, endoscopy and imaging techniques, such as MRI and CT, are the primary tools used to diagnose and assess CRC progression. Endoscopy is a procedure in which the gastrointestinal tract can be visualized and sampled. Colonoscopy has been shown to decrease CRC incidence by up to 76% and lower mortality by up to 65% [5,6,7]. However, it is an invasive technique that may result in low compliance. In contrast, CT and MRI are non-invasive but do not optimize cost and reliability [8,9]. Other non-invasive diagnostic measures such as the fecal occult blood test (FOBT) and carcinoembryonic antigen (CEA) blood test, have low sensitivity, ranging from 30% to 60% and cannot be used alone to detect or predict tumor progression [10,11,12,13].

One promising biomarker candidate of CRC is microRNA (miRNA). miRNAs are short 22 nucleotide sequences that inhibit gene expression and are dysregulated in cancer. miRNAs bind to mRNA to either degrade mRNA via the RNA-induced silencing complex (RISC), destabilize mRNA via decapping and deadenylation, or prevent translational initiation and elongation via ribosomal interactions [14]. Several miRNAs are dysregulated in cancer, including a group of miRNAs termed “oncomiRs”. The first oncomiR to be described was miR-21, a regulator of phosphatase and tensin homolog (PTEN) [15], programmed cell death 4 (PDCD4) [16], Sprouty 1 and 2 (SPRY1 and SPRY2) [17,18], and other tumor suppressors and transcriptional regulators [19]. In mice, miR-21 overexpression promoted tumorigenesis, while miR-21 knockout mice were protected against tumor formation [20]. Human studies have corroborated these findings, as elevated miR-21 levels have been associated with advanced tumor grade and poor prognosis across cancer types [21,22].

miR-20a is an oncomiR that participates in cell proliferation and cancer progression. Along with miR-17, miR-20b, miR-93, miR-106a, and miR-106b, miR-20a is a member of the miR-17 family, a polycistronic group of functionally related miRNAs that contain the same seed sequence [23,24]. The oncogene MYC induces the miR-17 family, which in turn dysregulates cell cycle progression, apoptosis, and tumor invasion via interactions with PTEN [25], E2F genes [26], and the transforming growth factor beta (TGF-β) pathway [27]. miR-20a has been shown to be upregulated across both solid and hematopoietic cancers [28] and has even been suggested as a diagnostic serum biomarker for gastric [29], nasopharyngeal [30], and prostate cancer [31]. However, miR-20a has been particularly noted for its regulatory role in CRC by upregulating the TGF-β signaling cascade. Previous in vitro experimentation has used dual luciferase assays to show direct miR-20a-mediated downregulation of homolog of drosophila mothers against decapentaplegic protein 2 and 4 (SMAD2 and SMAD4) [32,33], TGF-β receptor 2 (TGFBR2) [34], and cyclin dependent kinase inhibitor 1A (CDKN1A) [35]. Given its biological function, miR-20a has been measured in several CRC patient cohorts, but findings have been inconsistent.

We systemically review and quantitatively synthesize the evidence pertaining to miR-20a as a CRC biomarker. Specifically, we examine the diagnostic potential of miR-20a by quantifying its expression in feces, serum, and tumor tissue, as well as evaluate its sensitivity and specificity in CRC detection. In addition, we quantify the prognostic power of miR-20a in predicting the overall survival (OS) rates of CRC patients via hazard ratio (HR) meta-analysis. Due to its status as an oncomiR, miR-20a is hypothesized to be upregulated in cancerous tissue and is expected to detect CRC with high sensitivity. We also expect that higher miR-20a levels may correlate with poor prognosis, as miR-20a promotes tumor invasion and metastasis [36].

## 2. Methods

### 2.1. Study Selection Criteria

Studies that met all of the following criteria were included in the review-study design: randomized control trial (RCT), pre-post study, cross-sectional study, case-control study, and cohort study; study subjects: adults aged 18 years and above with colon, rectal, or CRC described by either tumor-node-metastasis (TNM) staging or pathology reports; main outcome: miR-20a expression in tumor, blood, or fecal samples or with at least one diagnostic or prognostic measure; article type: peer-reviewed publications; and language: English.

Studies were excluded from the review if they met any of the following criteria: non-English publications, reviews or case studies, and non-peer reviewed articles (e.g., dissertations or conference proceedings).

### 2.2. Search Strategy

A keyword search was performed in the PubMed and Web of Science databases. The search algorithm included all possible combinations of the keywords (with wildcard characters) from the following two groups: (1) “miR-20a-5p”, “mir-20a”, “microRNA-20a”, “has-mir-20a”, “mir20a”, “microRNA 20a”, and “mir 20a”; and (2) ‘‘colon cancer”, “rectal cancer”, and “colorectal cancer”. Titles and abstracts of the articles identified through the keyword search were screened against the study selection criteria. Potentially relevant articles were retrieved for evaluation of the full texts.

A cited reference search (i.e., forward reference search) and a reference list search (i.e., backward reference search) were conducted based on the articles from the keyword search. Articles identified through forward/backward reference search were further screened and evaluated using the same study selection criteria. The reference search was repeated on all newly-identified articles until no additional relevant article was found. The two authors of this review, Laura Moody and Svyatoslav Dvoretskiy, jointly determined the inclusion/exclusion of all articles retrieved in full texts and discrepancies were resolved through discussion.

### 2.3. Data Extraction

Relevant data were extracted according to the following general categories: author(s), publication year, study design, specimen type, the technical methodology used, sample size, and participant characteristics. Diagnostic and prognostic measures were also extracted. miR-20a expression was recorded as either a fold change, median ± standard deviation (SD), mean ± SD, or as simply up or down-regulated. The sensitivity and specificity of miR-20a as a diagnostic biomarker of CRC was denoted using receiver operating characteristic (ROC) curves. We extracted the area under the ROC curve (AUROC) along with a 95% confidence interval (CI). Prognostic measures included disease-free survival (DFS) time and OS time that was reported in Kaplan-Meier curves and HRs.

### 2.4. Meta-Analysis

A meta-analysis was performed to estimate the pooled effect size of miR-20a expression fold change, AUROC, and HR. Study heterogeneity was assessed using the I^2^ index. The level of heterogeneity represented by the I^2^ index was interpreted as modest (I^2^ ≤ 25%), moderate (25% < I^2^ ≤ 50%), substantial (50% < I^2^ ≤ 75%), or considerable (I^2^ > 75%). A fixed-effect model would be estimated when modest to moderate heterogeneity was present, and a random-effect model would be estimated when substantial to considerable heterogeneity was present. Publication bias was assessed by the Begg’s and Egger’s tests. All statistical analyses were conducted using the Stata 14.2 SE version (StataCorp, College Station, TX, USA). Specific STATA commands included “metan” and “metabias”. All analyses used two-sided tests, and *p*-values less than 0.05 were considered statistically significant. Summary AUROC curve was generated using R version 3.3.2 (R Foundation for Statistical Computing, Vienna, Austria).

### 2.5. Study Quality Assessment

The quality of all studies included in the review was evaluated by the following 10 quality assessment criteria adapted from Littell et al. [37]: (1) Was the research question clearly stated? (2) Were the inclusion and exclusion criteria clearly stated? (3) Were the subjects in the study representative of the pathological population? (4) Were the main findings of the study clearly described? (5) Was a control group included, and if so, did it consist of non-tumor specimens from healthy age- and gender-matched subjects? (6) Were diagnostic or prognostic measures clearly defined (e.g., TNM stage or five-year survival rate)? (7) Were samples collected from a relevant source (i.e., tumor tissue, blood, or feces) in a manner to prevent degradation and contamination? (8) Was miRNA expression measured by a validated technique (e.g., miRNA-seq, microarray or quantitative real-time PCR [q-PCR])? (9) Was a sample size justification via power analysis provided? (10) Were potential confounders properly controlled in the analysis? Each of the 10 criteria was scored on a scale of zero to two, depending on whether the criterion was unmentioned or unmet (0), partially met (1), or completely met (2). The possible total score ranged from zero to 20. The two authors of the review, Laura Moody and Svyatoslav Dvoretskiy, independently scored each study and discussed any disagreement. The study quality score was used to quantify the strength of existing evidence but was not used in the study selection. 

## 3. Results

### 3.1. Study Selection

**Figure 1:**
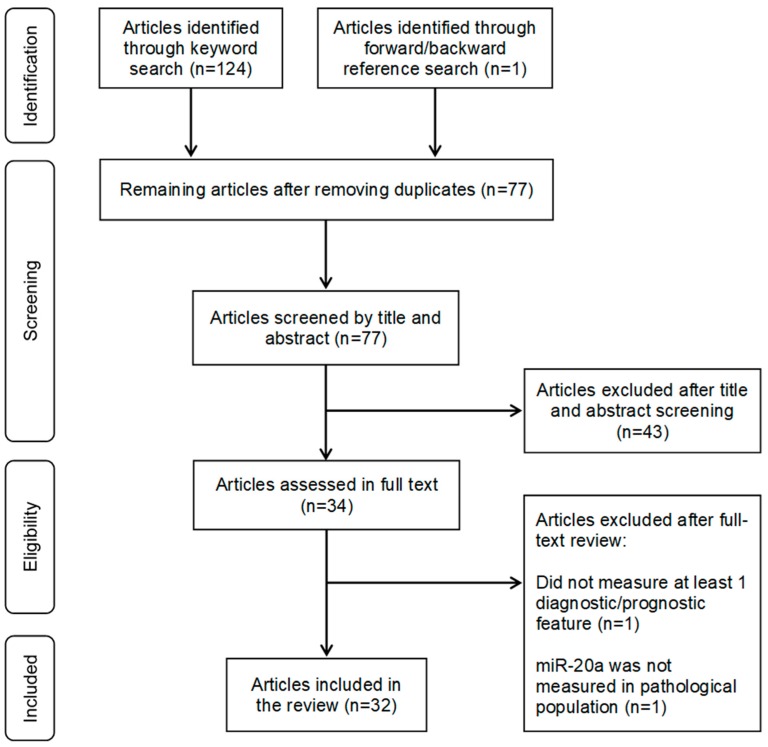
Flowchart of the literature search and selection process of eligible studies.

As shown in Figure 1, among 77 total unduplicated articles identified through the keyword and reference search, 43 were excluded in the title and abstract screening. The full texts of the remaining 34 articles were reviewed, and two were excluded-one did not provide a diagnostic or prognostic measurement (*n* = 1) [38], and one did not measure miR-20a in the tissue of interest in the pathological population (*n* = 1) [39]. The remaining 32 articles were included in the review [32,40,41,42,43,44,45,46,47,48,49,50,51,52,53,54,55,56,57,58,59,60,61,62,63,64,65,66,67,68,69].

### 3.2. Basic Characteristics of The Selected Studies

Table 1 summarizes the 32 studies included in the systematic review. The studies were conducted in China (*n* = 10) [32,45,46,49,62,63,66,68,69], the United States (*n* = 7) [40,42,54,55,58,61,65], Japan (*n* = 4) [48,52,59,64], Germany (*n* = 3) [41,50,56], Spain (*n* = 3) [43,44,60], Italy (*n* = 2) [51,57], Egypt (*n* = 1) [67], the Netherlands (*n* = 1) [47], and Turkey (*n* = 1) [53]. All studies adopted an observational design and were either case-control (*n* = 22) [40,42,43,45,46,47,49,50,52,53,54,55,57,59,61,62,63,64,65,66,67,69] or cohort studies (*n* = 10) [32,41,44,48,51,58,60,68]. Patients represented all stages of colon and rectal cancer. Sample size was widely variable between studies, with a mean of 155, a median of 68, and a range from three to 1141. Across all studies, 5014 cancer and 2863 control cases were analyzed. Females accounted for 18.2% to 57.6% of the study samples. The type of biological specimen collected included feces (*n* = 3) [40,57,66], serum (*n* = 8) [41,44,46,49,50,64,67,68], and tumor (*n* = 25) [32,42,43,44,46,47,48,49,51,52,53,54,55,56,57,58,59,60,61,62,63,65,66,68,69]. All studies used q-PCR or microarray techniques to measure the relative expression of miRNAs. Reported diagnostic or prognostic features included expression of miR-20a (E, *n* = 22) [40,42,43,45,46,47,49,50,52,53,54,55,57,58,59,61,62,63,64,66,67,68], AUROC (*n* = 4) [45,50,66,67], disease free survival (DFS, *n* = 2) [32,60], and overall survival (OS, *n* = 5) [32,46,58,60,68].

### 3.3. miR-20a as a Diagnostic Biomarker of CRC

As Table 2 reports, 22 of the 32 studies reported a directional change of miR-20a in either feces, serum, or tumor tissue among CRC patients. Twenty-one studies [40,42,43,45,46,47,49,50,52,53,54,55,57,58,59,62,63,66,67,68] found that miR-20a was upregulated in cancer specimens. However, this upregulation was statistically significant in only 16 studies [45,46,47,49,50,52,53,55,57,58,59,61,63,66,67,68]. One study reported a downregulation of miR-20a in the serum of CRC patients, but this change was statistically nonsignificant [64]. Sample-size-weighted fold increases of 1.99 (SD = 0.65), 1.74 (SD = 0.68) and 2.86 (SD = 2.61) in miR-20a were identified in the feces [57,66], serum [43,45,49,64,67], and tissues of cancer patients (compared to the healthy controls) [42,46,52,53,54,55,58,62], respectively. Across biological samples, there was an overall 2.35 (SD = 1.89) weighted fold increase of miR-20a in CRC patients versus controls.

**Figure 2:**
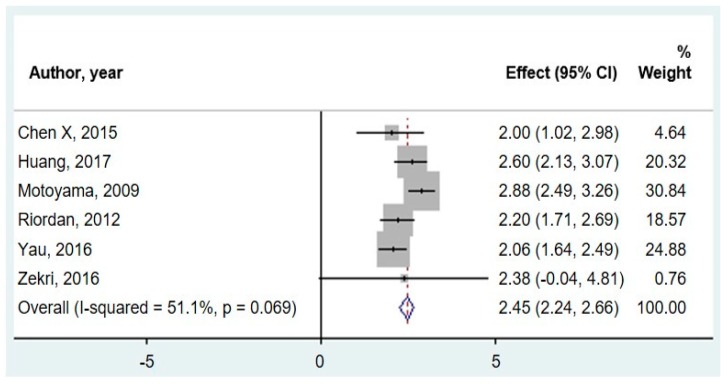
Forest plot for the meta-analysis of miR-20a expression fold change in CRC versus controls across tissue types.

Figure 2 shows the meta-analysis of miR-20a expression in CRC versus control samples. Only six studies reported adequate data to be included in the meta-analysis. Fold change data were extracted from the six relevant studies and evaluated using a random-effect model. One study measured fecal samples [66], two measured serum [49,67], and three measured tumor tissue [46,52,55]. A pooled fold change estimation of 2.45 (95% CI: 2.24–2.66) was calculated in CRC versus controls.

**Figure 3:**
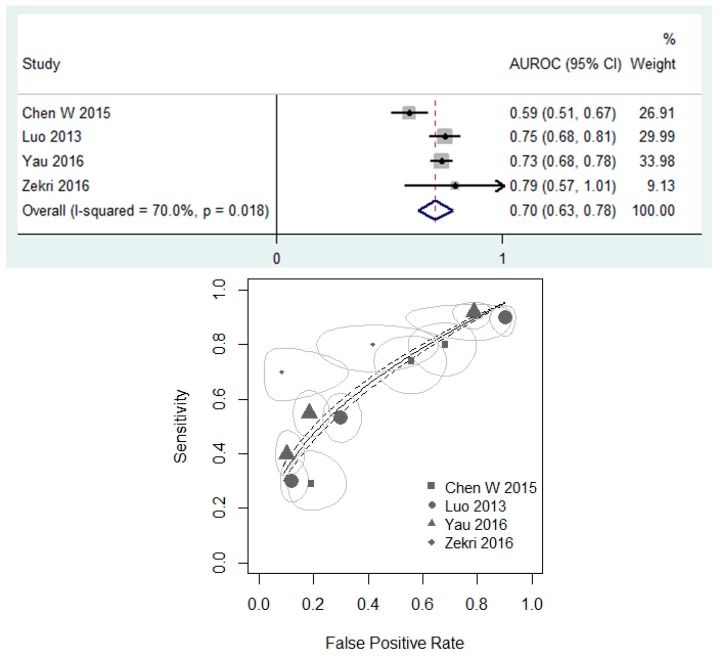
(Top) Forest plot for meta-analysis of AUROC using random effects analysis. (Bottom) Summary ROC curve. ROC curve and standard error are presented. The size of the points is representative of the weight of the individual studies.

Figure 3 assesses the performance of miR-20a as a biomarker for CRC detection using the AUROC. In the four relevant studies [45,50,66,67], the estimated AUROC ranged from 0.59 [45] to 0.79 [67]. Three studies examined circulating miRNA [45,50,67], and one focused on fecal expression [66]. Three studies examined miR-20a as an independent predictor of CRC [45,66,67], and one study considered miR-20a as part of 12 miRNA diagnostic panels [50]. The random-effect model reported a pooled AUROC of 0.70 (95% CI: 0.63–0.78).

### 3.4. mir-20a as a Prognostic Biomarker of CRC Survival

**Figure 4:**
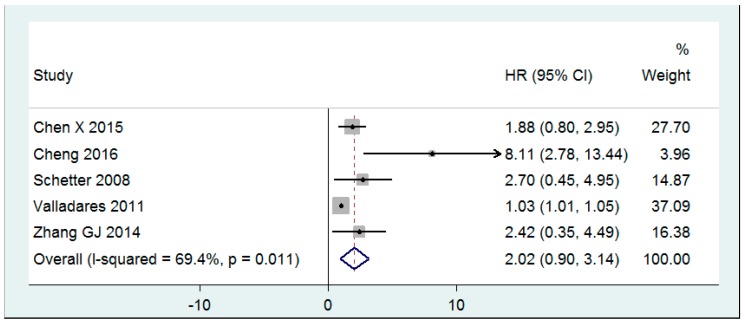
Forest plot for the meta-analysis of HR for OS evaluated by random effects analysis.

Figure 4 shows the prognostic value of high miR-20a expression reported as the estimated HR for OS. Patient outcome based on miR-20a levels was examined in five articles [32,46,58,60,68]. Two studies [32,60] reported DFS, and all five studies reported the OS. Findings were presented in a Kaplan-Meier curve with an HR and 95% CI. Splitting the dataset into high and low miR-20a expression groups was achieved by using the highest tertile [58], the median [68], a software tool [46,60], or was not disclosed [32]. In patients with high miR-20a expression, the two reported HRs for DFS were 1.02 (95% CI: 1.00–1.04) [60] and 7.90 (95% CI: 4.3–14.53) [32]. Similarly, all five studies reported a statistically significant HR for an OS greater than one for patients with high miR-20a levels. HR values ranged from 1.03 [60] to 8.11 [32]. A random-effect model reported a pooled HR for an OS of 2.02 (95% CI: 0.90–3.14).

No publication bias was identified, as neither Egger’s tests nor Begg’s tests were statistically significant for any of the outcomes.

### 3.5. Study Quality Assessment

Table 3 shows the study quality assessment. Scores ranged between 12 [47] and 18 [42] with an average score of 14.94 ± 1.48. Nearly all studies scored high on the clarity of the research question (1.92 ± 0.19) and the technique used to quantify miRNA (2.00 ± 0.00). All studies used a microarray or qPCR for miRNA quantification. In addition, appropriate sample collection (1.85 ± 0.23) was met at least partially by all studies, as all collected relevant specimen types and most documented the collection procedures in detail. The lowest scoring category was the employment of a power analysis (0.10 ± 0.29). Sample size justification via power analysis was conducted by only two studies [42,64].

## 4. Discussion

This study synthesized existing evidence on miR-20a as a diagnostic and prognostic biomarker of CRC. Diagnostic and prognostic efficacy was examined in a total of 5014 CRC patients in 32 studies. Overall, miR-20a was found to be a potentially promising diagnostic marker of CRC. In feces, serum, and tumor tissue, a majority of the evaluated studies found an upregulation of miR-20a. On the other hand, high miR-20a expression was not a statistically significant predictor of poor patient prognosis.

In all biological specimen, miR-20a was found to be more highly expressed in cancer patients than in control subjects. These findings are consistent with mechanistic studies that found miR-20a to target numerous genes involved in cell cycle regulation. Tumorigenesis is initially characterized by the inadequacy of DNA repair machinery to maintain appropriate cellular function. Downregulation of tumor suppressor genes and activation of oncogenes leads to a death-resistant, hyperplasic state that results in cancer progression and eventually metastasis. miR-20a is a member of the miR-17 family known for its oncogenic properties. Indeed, miR-20a overexpression in cell lines has been shown to promote cell cycle progression [35], while inhibition of miR-20a induces an E2F1-associated DNA damage response and G1 checkpoint activation [70]. Thus, it is likely that the high levels of miR-20a observed in the meta-analysis reflect an inability to check cellular growth, resulting in a malignant state.

The potential of miR-20a as a positive diagnostic biomarker of CRC was further supported by evaluating sensitivity and specificity through the AUROC analysis. While the gold-standard for CRC diagnosis remains colonoscopy, non-invasive tests have been considered for screening at-risk patients. Carcinoembryonic antigen (CEA) and cancer antigen 19-9 (CA19-9) are two such blood tests that have already been implemented in the clinic. One meta-analysis evaluated the use of CEA in detecting CRC recurrence and found an AUROC of 0.75 [71]. Similarly, studies examining the diagnostic validity of CA19-9 have observed AUROC values between 0.69 and 0.77 [72,73]. Our meta-analysis reported a pooled AUROC of 0.70 (95% CI: 0.63–0.78). This value is comparable to those of CEA and CA19-9, suggesting that miR-20a may be useful in CRC diagnosis.

In the current review, CRC prognosis was assessed through HRs for OS. All studies reported statistically significant HR values of greater than one for both DFS and OS, but only two studies evaluated DFS, so a meta-analysis was not performed. Five studies were pooled for the meta-analysis of HR for OS. Results suggest that patients with high miR-20a expression die earlier than those with lower expression. While the effect size was large, it was not significantly greater than one, so we cannot conclude that high miR-20a expression is indicative of lower OS. Previous literature has quantified the prognostic power of other RNAs in CRC. Two meta-analyses found miR-21 to be a predictor of poor prognosis [74,75]. Beyond miRNA, the prognostic value of mRNA expression has also been explored in CRC patients. For instance, overexpression of the metastasis-associated in the colon cancer 1 (MACC1) gene resulted in poor survival prognosis [76], while amphiregulin (AREG) and epiregulin (EREG) genes were found to be favorable prognostic biomarkers [77]. Compared to other RNA prognostic predictors, the pooled HR for OS of miR-20a calculated in the present review was relatively high. However, possibly due to the small number of studies, our modeling results were statistically nonsignificant. In order to determine the efficacy of miR-20a as a prognostic biomarker of CRC, further clinical investigation should be performed.

The present meta-analysis had limitations. Only a few studies specifically examined miR-20a in the prognosis and diagnosis of CRC. In addition, some studies did not report sufficient data to be included in the meta-analysis. Much of the data was reported without standard deviation or the necessary information (e.g., CI) to calculate the standard deviation. Several studies focused on multiple miRNAs rather than miR-20a alone, and data were often presented as a ratio of one miRNA to another. Thus, only a few studies clearly compared between cancer and control. This compromised the statistical power to identify the effect of miR-20a as a diagnostic and prognostic biomarker of CRC. Furthermore, many studies did not make a distinction between colon and rectal cancer and did not report tumor location. Given the differences in genetic profile and clinical outcomes between colon and rectal cancer, as well as between proximal and distal colon carcinomas [78,79,80], it is unclear whether miR-20a could be a better biomarker in specific tumor subtypes. Finally, there still exist several barriers to interpret and implement miRNA biomarkers in the clinic. The constantly evolving methodology has made it difficult to compare between studies, especially in establishing a baseline miRNA expression level. Genome-wide next-generation sequencing allows researchers to simultaneously evaluate multiple miRNAs and has great potential for biomarker discovery, but a standardized procedure has yet to be developed for quantifying miRNA and drawing clinically-relevant conclusions. Proper expertise in both data analysis and cancer biology is important for separating signal from noise when identifying CRC biomarkers.

## 5. Conclusions

In conclusion, this study systematically synthesized and quantitatively analyzed the scientific evidence on miR-20a as a diagnostic and prognostic biomarker in CRC. The meta-analysis uncovered elevated miR-20a levels in fecal, serum, and tumor samples of CRC patients and supported the hypothesis that miR-20a is a sensitive diagnostic tool. Contrary to expectations, the estimated HR for OS showed that higher miR-20a expression was not indicative of a poor survival prognosis. Clinicians interested in improving CRC detection may consider miR-20a as a potentially useful diagnostic biomarker.

## Figures and Tables

**Table 1:** Basic characteristics of included studies. CC, case control; COH, cohort; C, colon; R, rectum; CR, colorectal; NR, not reported; F, feces; T, tumor; S, serum; E, miR-20a expression; OS, overall survival; DFS, disease-free survival; AUROC, area under the receiver operating characteristic curve.

Author/Year	Study Design	Cancer Pathology	TNM Stage	Biological Specimen	Sample Size (Cancer, Control)	% Female (Cancer, Control)	Measurement Method	Reported Value(s)
*Ahmed 2013*	*CC*	*C*	I–IV	F	40, 20	42.5, 50	*Microarray, qPCR*	E
*Azizian 2015*	*COH*	*R*	NR	S	42, 0	40.5, NA	*qPCR*	*--*
*Bovell 2013*	*CC*	*CR*	I–IV	T	381, 381	50.7, 50.7	*qPCR*	*E*
*Brunet 2013*	*CC*	*CR*	III–IV	S, T	42, 26	35.7, 50	*Microarray*	*E*
*Caritg 2016*	*COH*	*C*	III–IV	T	69, 0	37.7, NA	*qPCR*	*--*
*Chen X 2015*	*CC*	*CR*	II–III	T	11, 11	18.2, 18.2	*qPCR*	*E, OS*
*Chen W 2015*	*CC*	*CR*	I–IV	S	100, 79	40, 44.9	*qPCR*	AUROC, E
*Cheng 2016*	*COH*	*CR*	I–IV	T	544, 0	44.9, NA	*qPCR*	*DFS, OS*
*de Groen 2015*	*CC*	*CR*	NR	T	52, 48	NR	*qPCR*	*E*
*Hotchi 2013*	*COH*	*R*	III–IV	T	43, 0	27.9, NA	*qPCR*	*--*
*Huang 2017*	*CC*	*CR*	NR	S, T	70, 70	NR	*qPCR*	*E*
*Luo 2013*	*CC*	*CR*	I–IV	S	180, 244	43.3, 57	*qPCR*	AUROC, E
*Molinari 2016*	*COH*	*R*	II–IV	T	108, 0	30.6, NA	*qPCR*	*--*
*Motoyama 2009*	*CC*	*CR*	I–III	T	67, 67	NR	*qPCR*	*E*
*Ozcan 2016*	*CC*	*CR*	II–IV	T	54, 42	37, NR	*qPCR*	*E*
*Pellatt 2016*	*CC*	*CR*	NR	T	1141, 812	45.2, 45.7	*qPCR*	*E*
*Riordan 2012*	*CC*	*R*	I–III	T	17, 17	NR	*qPCR*	*E*
*Ristau 2014*	*COH*	*CR*	I–IV	T	35, 0	40, NA	*qPCR*	*--*
*Rotelli 2015*	*CC*	*CR*	I–III	F, T	20, 20	35, NR	*qPCR*	*E*
*Schetter 2008*	*COH*	*C*	I–IV	T	197, 197	38.1, 38.1	*Microarray, qPCR*	*E, OS*
*Tsuchida 2011*	*CC*	*C*	I–IV	T	13, 9	NR	*qPCR*	*E*
*Valladares 2011*	*COH*	*CR*	I–IV	T	38, 0	34.2, NA	*qPCR*	*DFS, OS*
*Volinia 2006*	*CC*	*C*	NR	T	46, 8	NR	*Microarray*	*E*
*Wang 2010*	*CC*	*CR*	NR	T	3, 3	NR	*Microarray*	*E*
*Xu 2015*	*CC*	*CR*	NR	T	30, 30	36.7, 36.7	*qPCR*	*E*
*Yamada 2015*	*CC*	*CR*	NR	S	160, 77	33.8, 45.5	*qPCR*	*E*
*Yantiss 2009*	*CC*	*CR*	I–IV	T	69, 0	44.9, NA	*qPCR*	*--*
*Yau 2016*	*CC*	*CR*	I–IV	F, T	198, 198	41.1, 57.6	*qPCR*	AUROC, E
*Zekri 2016*	*CC*	*CR*	NR	S	130, 24	38.5, 37.5	*Microarray*	AUROC, E
*Zhang GJ 2014*	*COH*	*CR*	I–IV	T	86, 86	38.4, 38.4	*qPCR*	*E, OS*
*Zhang J 2014*	*COH*	*CR*	III–IV	S	253, 0	44.7, 0	*Microarray*	*--*
*Zhang JX 2013*	*CC*	*C*	II–IV	T	735, 735	41.3, 41.3	*Microarray*	*--*

**Table 2:** miR-20a expression in colorectal cancer (CRC) versus controls. Up, higher expression in CRC; down, lower expression in CRC; NR, not reported.

Author/Year	miR-20a (Expression in CRC vs. Control)	Fold Change (CRC vs. Control)	*p*-Value	CRC Patients
**Feces**				
Ahmed 2013	up	NR	NR	40
Rotelli 2015	up	1.1461	*p* < 0.0001	20
Yau 2016	up	2.063	*p* = 0.0065	198
**Feces weighted fold change ± SD: 1.99 ± 0.65**				
**Serum**				
Brunet 2013	up	1.378	*p* = 0.593	42
Chen W 2015	up	1.64	*p* = 0.038	100
Huang 2017	up	2.6	*p* < 0.05	70
Luo 2013	up	NR	*p* = 0.001	180
Yamada 2015	down	0.98	*p* = 0.834	160
Zekri 2016	up	2.385	*p* = 0.017565	130
**Serum weighted fold change ± SD: 1.73 ± 0.68**				
**Tumor**				
Bovell 2013	up	2.05	NR	345
Chen X 2015	up	2	*p* = 0.004	11
de Groen 2015	up	NR	*p* < 0.01	52
Motoyama 2009	up	2.875	*p* < 0.05	67
Ozcan 2016	up	8.9509	*p* = 0.002625	54
Pellatt 2016	up	3.765	NR	1141
Riordan 2012	up	2.2	*p* < 0.00001	17
Schetter 2008	up	2.3	*p* < 0.001	197
Tsuchida 2011	up	NR	*p* < 0.05	13
Volinia 2006	up	NR	*p* = 0.0025	46
Wang 2010	up	2.41	NR	3
Xu 2015	up	NR	*p* < 0.001	30
Zhang GJ 2014	up	NR	*p* < 0.001	86
**Tumor weighted fold change ± SD: 2.86 ± 2.61**				
**Overall weighted fold change ± SD: 2.35 ± 1.89**				

**Table 3:** Study quality assessment.

Criterion	Mean	SD
1. Was the research question clearly stated?	1.92	0.27
2. Were the inclusion and exclusion criteria clearly stated?	1.44	0.71
3. Were the subjects in the study representative of the pathological population?	1.68	0.59
4. Were the main findings of the study clearly described?	1.77	0.46
5. Was a control group included? Did it consist of the non-tumor specimen from healthy age- and gender-matched subjects?	1.78	0.49
6. Were diagnostic or prognostic measures clearly defined (e.g., TNM stage, five-year survival rate, etc.)	1.27	0.70
7. Were samples collected from a relevant source (tumor tissue, blood, or feces) in a manner to prevent degradation and contamination?	1.81	0.39
8. Was miRNA expression measured with a conventional, well-validated technique (e.g., miRNA-seq, microarray, qPCR, etc.)?	2.00	0.00
9. Was a sample size justification via power analysis provided?	0.13	0.45
10. Were potential confounders properly controlled in the analysis?	1.27	0.45
Total Score	15.06	1.87
